# Public Health Decision-Making in the Real World: Four Points to Reshape It After COVID-19

**DOI:** 10.1017/dmp.2020.108

**Published:** 2020-04-21

**Authors:** Emanuele Torri, Giandomenico Nollo

**Affiliations:** Department of Health and Social Policies, Autonomous Province of Trento, Trento, Italy; Department of Industrial Engineering - BIOtech Labs, University of Trento, Trento, Italy

**Keywords:** public health practice, public health, quality of health care, risk management, safety management

The coronavirus disease 2019 (COVID-19) outbreak is rapidly progressing worldwide, tremendously impacting health systems, science, and society.^1^ Uncertainty is unparalleled in living memory, and involves the urgency of deciding with limited resources and under pressures from conflicting interests. Drawing from the hard lessons of Italy’s experience, the European country most affected by the pandemics to date,^2^ we wish to share ideas to improve emergency decision-making.

## GOVERNANCE

Expanding international coordination^3^ and health-care accreditation programs to guarantee reliability of hygiene practices, surveillance, and safety systems on the ground is crucial now. A special attention toward strengthening education for leadership development of public health officials and clinicians is needed to enable engagement, resilience, and trust.

## DATA-DRIVEN MANAGEMENT

Information and data sets for COVID-19 research should be in the public domain, to provide broader development of models and data supporting health authorities and agencies in public decisions. Peer observations and bottom-up inputs should be collected. Multiple scientific competence centers should be institutionalized as “scientific civil protection” for activations during emergencies to perform timely analysis and experiments tailored on policy questions.

## HEALTH TECHNOLOGY ASSESSMENT

Context-based analysis of cultural, organizational, economic, social, and ethical issues is vital given the complexity of public health. First, politicians and the public should be thoroughly educated in health technology assessment. Second, we need practical models to expedite the appraisal process and resolve technological issues in emergencies. Third, in addition to the opinion of a few experts, we could exploit the value of collective knowledge and crowdsourcing data within structured consensus mechanisms.

## CLINICAL GUIDELINES AND PROTOCOLS

With limited availability of evidence and the need for broad consensus meetings, relying only on eminences or slow bureaucratic orders is not the best choice. Instead, it is essential to prompt researchers and scientists to fully review “expert evidence”^4^ by basing decisional frameworks on practical insights of stakeholders and frame recommendations as actionable points disseminated through multiple channels.

While a wave of changes in culture, technology, and practice is sweeping the globe,^5^ we have an unprecedented opportunity for optimizing the compromise between science and real-word decisions.
